# Maximal cardiopulmonary exercise testing in glioblastoma patients undergoing chemotherapy: assessment of feasibility, safety, and physical fitness status

**DOI:** 10.1007/s11060-024-04629-y

**Published:** 2024-04-01

**Authors:** Johanna Jost, Klaus Völker, Ralf Brandt, Walter Stummer, Steffi Urbschat, Ralf Ketter, Dorothee Wiewrodt, Rainer Wiewrodt, for the MMH Trial Investigators, Maren Kloss, Nora Hansel, Irmtraud Früchte, Ross Julian, Lothar Thorwesten, Joachim Gerß, Andreas Faldum, Joachim Oertel, Philipp Lepper, Kathleen Jetschke, Sylvia Rekowski, Carolin Weiss Lucas, Sophia Kochs, Freerk Baumann

**Affiliations:** 1 Department of Neurosurgery, University Hospital, University Münster, Münster, Germany; 2 Institute of Sports Science, University Hospital, University Münster, Münster, Germany; 3 Department of Neurosurgery, University Hospital, Saarland University, Saarbrücken, Germany; 4 Pulmonary Research Division, Department of Medicine A, University Hospital, University Münster, Münster, Germany

**Keywords:** Cardiopulmonary Exercise Testing (CPET), Glioblastoma, Self-reported exercise, Cardiorespiratory fitness, Active-in-Neuro-Oncology (ActiNO)

## Abstract

**Purpose:**

Maximal cardiopulmonary exercise testing (max. CPET) provides the most accurate measurement of cardiorespiratory fitness. However, glioblastoma (GBM) patients often undergo less intensive tests, e.g., 6-min walk test or self-rating scales. This study aims to demonstrate feasibility and safety of max. CPET in GBM patients, concurrently evaluating their physical fitness status.

**Methods:**

Newly diagnosed GBM patients undergoing adjuvant chemotherapy were offered participation in an exercise program. At baseline, max. CPET assessed cardiorespiratory fitness including peak oxygen consumption (VO_2_peak), peak workload, and physical work capacity (PWC) at 75% of age-adjusted maximal heart rate (HR). Criteria for peak workload were predefined based on threshold values in HR, respiratory quotient, respiratory equivalent, lactate, and rate of perceived effort. Data were compared to normative values. Adverse events were categorized according to standardized international criteria. Further, self-reported exercise data pre- and post-diagnosis were gathered.

**Results:**

All 36 patients (median-aged 60; 21 men) met the predefined criteria for peak workload. Mean absolute VO_2_peak was 1750 ± 529 ml/min, peak workload averaged 130 ± 43 W, and mean PWC was 0.99 ± 0.38 W/kg BW, all clinically meaningful lower than age- and sex-predicted normative values (87%, 79%, 90%, resp.). Only once (3%) a minor, transient side effect occurred (post-test dizziness, no intervention needed). Self-reported exercise decreased from 15.8 MET-h/week pre-diagnosis to 7.2 MET-h/week post-diagnosis.

**Conclusion:**

Max. CPET in this well-defined population proved feasible and safe. GBM patients exhibit reduced cardiorespiratory fitness, indicating the need for tailored exercise to enhance health and quality of life. CPET could be essential in establishing precise exercise guidelines.

**Supplementary Information:**

The online version contains supplementary material available at 10.1007/s11060-024-04629-y.

## Introduction

According to the World Health Organization (WHO), glioblastoma multiforme (GBM) represents the most malignant form of intrinsic brain tumor. Despite multimodal therapies, prognosis remains poor [1]. Many GBM patients suffer neurocognitive, functional, and emotional impairments, diminishing their quality of life (QoL) [2]. The primary objective of all treatment endeavors is to extend life while maximizing QoL. Supportive therapies are well established to improve QoL in cancer patients [3], including physical activity (PA) [4].

Previous studies on exercise programs in glioma patients indicate positive impacts primarily on symptom burden [5]. However, it is worth noting that cautionary guidelines for brain tumor patients persist, e.g., in a guide related to “exercise in cancer”, there is a clear warning for brain tumor patients against intensive exercise [6]. The guide emphasizes potential risks of neurological episodes, epilepsy, or even sudden unconsciousness for these patients. Further, limited studies, especially in GBM, hinder definitive exercise guidelines. To address this gap we aimed to accurately create and evaluate an exercise intervention program in GBM, building upon our experience spanning over a decade with training brain tumor patients [7] with various grades and treatment regimens. In this retrospective analysis, we demonstrated the feasibility and safety of intensive exercise in GBM patients who had undergone surgery and chemoradiation, and who concomitantly received adjuvant chemotherapy [7].

However, there are no prospective data and these participants were not subjected to maximum cardiopulmonary exercise testing (max. CPET; including, among other parameters, peak oxygen consumption (VO_2_peak) assessment), which is considered the gold standard, and offers numerous benefits [8]. First, it provides crucial information about a person's current physical condition (i.e., cardiorespiratory fitness, VO_2_peak, and anaerobic threshold), making it a foundation for developing individualized exercise plans. Second, CPET can help identify and address significant comorbidities, which are especially important in conditions like GBM, since median-aged patients of 60 years are more likely to have coexisting medical conditions [9]. Third, CPET motivates individuals by providing clear progress tracking from the first measurement. In oncology generally, CPET has already proven to be a valuable tool and has been safely performed in other tumor entities, including breast, lung, head and neck, and prostate cancer [10–13].

In contrast, evidence to use max. CPET to assess training programs in GBM is vague, likely due to concerns about overexertion and potential harm, i.e., epilepsy. Therefore, this study aims to systematically and prospectively assess max. CPET in GBM undergoing adjuvant treatment to prove its feasibility and safety. Furthermore, we aim to present the physical fitness status of GBM patients after completion of chemoradiation, comparing it to normative data, analyzing their CPET results, and examining how their activity behavior has changed following diagnosis.

## Methods

### Design and procedure

We report on the results of max. CPET in patients with GBM undergoing chemotherapy. The data presented in this paper were collected as part of the baseline assessment of an ongoing prospective study in malignant glioma (ClinicalTrials NCT05015543) that aims to investigate the impact of a 16-week exercise program “ActiNO” [7] primarily on the physical performance in this patient population (abbreviated “MMH”: **M**obil **M**it **H**irntumor (German); English: active with brain tumor). The study was approved by the local ethics committee (file number: 2015–087-f-S). All patients provided written informed consent before starting the study.

### Participants

Between July 2020 and March 2023, all patients who were consecutively enrolled and recruited at the University Hospital Münster, Department of Neurosurgery as part of the MMH study were included in the analysis. Major inclusion criteria were: newly diagnosed GBM, Karnofsky Performance Status (KPS) ≥ 70, age ≥ 18 years, completion of surgical therapy, completion of combined chemoradiation, ongoing adjuvant chemotherapy, thrombocytes > 50,000/µl, Hemoglobin > 8 mg/dl, ability to give written consent, and very good German language skills. Major exclusion criteria included strong, permanent pain restricting movement, impairment of consciousness, acute infection, fever, pregnancy and/or lactation, contraindications to CPET, and insufficiently controlled epilepsy despite anticonvulsive therapy (defined as > 3 focal seizures per day or > 1 generalized seizure in the previous 3 days). Clinical data were extracted from the patients’ charts, and sociodemographic data were collected through a questionnaire.

### Self-reported exercise

Information on patients’ PA levels were collected once as part of the screening procedure through a self-designed, non-validated, questionnaire (Suppl. Figure 1). Its purpose was to obtain an estimation from patients’ regarding their PA behavior. Patients were asked to retrospectively assess their activity levels both before diagnosis and at the current time. Information on self-reported exercise included type, frequency, and duration of PAs performed. Based on these details, the metabolic equivalents (METs) in hours per week were calculated for each patient. METs are a recognized measure utilized in exercise physiology to quantify the intensity of various PAs. They provide a standardized method for comparing the energy expenditure of different activities relative to the resting metabolic rate (resting equals relaxed sitting, which accounts for 1 MET). A table detailing the METs assigned to each of the PAs reported by the patients is included in the Supplement (Suppl. Table 4): 3 METs were assigned to an hour spent in mild-intensity PAs, 5 METs to an hour in moderate-intensity PAs, and 7 METs to an hour in vigorous-intensity PAs.

### Cardiopulmonary Exercise Test (CPET)

**Fig. 1:**
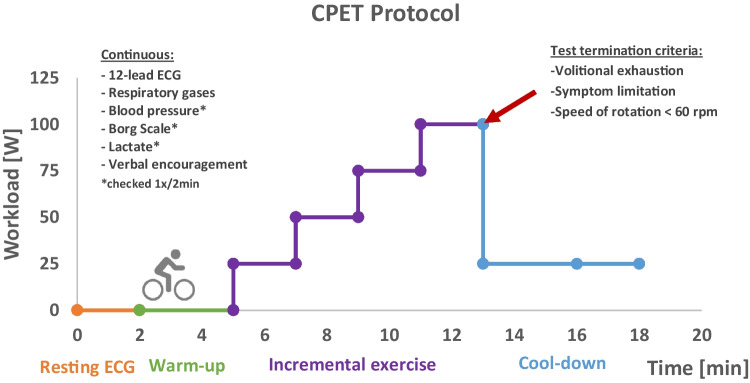
Cardiopulmonary exercise testing procedure. The protocol began with an initial resting electrocardiogram (ECG, 12-lead) recording, followed by a 3-min warm-up period at 0 watts. After warm-up, the exercise test commenced at an initial workload of 25 watts. Each stage of the exercise test was increased by 25 watts every 2 minutes until the participants reached their maximal capacity (volitional exhaustion or symptom limitation). During the exercise test, participants were verbally encouraged by the test administrators. The patients were instructed to maintain a constant speed between 60 and 80 rpm. Following the completion of the exercise test, a 5-min cool-down phase was performed at a workload of 25 watts

To evaluate the cardiorespiratory fitness of all participants, an incremental physician-monitored exercise test using spiroergometry was conducted on an upright bicycle ergometer (CareFusion, Höchberg, Germany) following guidelines published by the WHO [14]. For a detailed description, refer to Fig. 1. To examine the hypothesis derived from our previous investigation, the protocol utilized in the present study closely resembled the methodology employed in our previous report (e.g., 25 Watt increments) [7], albeit with some distinctions. Due to the absence of a spiroergometric system in our previous study, the pulse-related power in watts at 75% of age-adjusted HRmax, known as Physical Work Capacity (PWC) assessed patients’ physical performance. In contrast, the current study additionally used spiroergometry to allow for more sophisticated measurements, i.e., VO_2_peak analysis.

### Feasibility and Safety

We used a combination of criteria, including heart rate (HR) (≥ 80% of norm), Borg scale (≥ 17), lactate levels (> 5 mmol/L), respiratory equivalent (> 35), and respiratory exchange ratio (RER, > 1.10), as summarized by Löllgen and Leyk [15] and Kroidl et al. [16], to determine if participants reached their maximal physical exertion. Peak performance was determined to be achieved if at least one of these criteria was exceeded. Adverse events were categorized according to the Common Terminology Criteria for Adverse Events (CTCAE), version 5.0.

### CPET pre-testing procedures

Prior to spiroergometric testing, all participants were provided with a comprehensive explanation of the testing procedures by a board-certified internist and pulmonary physician accompanying the whole examination. Informed consent was obtained from each participant. The patients were instructed to continue taking their regular medications, however, special care was taken to ensure that no chemotherapy was administered on the day of the test. Before each measurement, calibration was conducted according to the guidelines of the certified spiroergometric system (MasterScreen™ CPX, CareFusion, Höchberg, Germany). This calibration process included volume calibration (high flow and low flow) as well as gas calibration for O_2_ and CO_2_. The entire procedure was performed by an experienced medical technical assistant, and under the supervision of the attending pulmonary physician.

#### Measured and calculated parameters

Continuous monitoring of HR was enabled through ECG, and respiratory gases were measured continuously (breath-by-breath). The highest 30-second values of all recorded cardiopulmonary function data were collected for analysis. Lactate levels were measured at each stage of exercise testing, while blood gas analysis was conducted prior to testing at rest and at maximum exertion. Blood pressure was measured 30 s prior to completion of each workload. At the same time, the Borg scale was employed to gauge perceived exertion. To assess participants' physical fitness in comparison to normative values, measured values of VO_2_peak and maximal workload were compared against published equations tailored to participants' sex, age, weight, and height, as outlined in the SHIP-study [17]. We selected the established equations proposed by the SHIP-study as our set-up exhibits great similarities (i.e., seated ergometer tests with an incremental CPET protocol, participants up to the age of 84). Additional information regarding the SHIP-study can be found in the Supplement (abstract, a table detailing population characteristics (Suppl. Table 1), and a table presenting reference value equations (Suppl. Table 2)). Moreover, the present study involved PWC using the same procedure as described in a previous paper [7].

### Statistical analyses

Standard descriptive analyses were executed, utilizing absolute and relative frequencies for categorical variables. For normally distributed data, means and standard deviations (SD) were presented, while medians and ranges or interquartile ranges (IQRs) were presented for non-normally distributed data. The Shapiro–Wilk test was used to assess the normality of the data. Physical outcomes were compared to predicted normative values using paired t-tests (normally distributed) or Mann–Whitney U test (non-normally distributed). For assessing correlations, we used Spearman rank correlation. The data were analyzed using SPSS 29.0 (IBM, Armonk, NY, USA).

## Results

### Participants

**Fig. 2:**
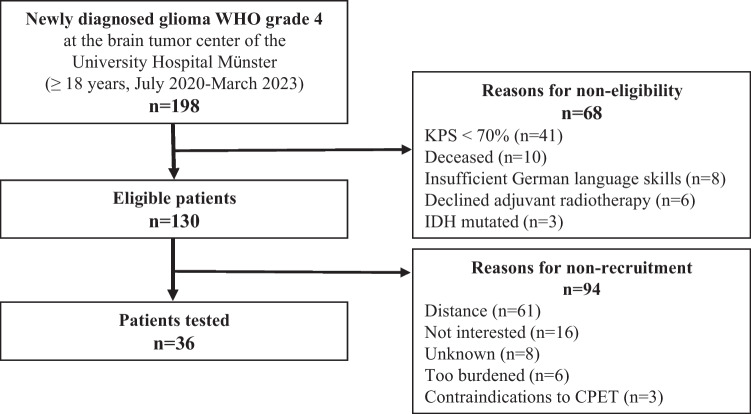
Recruitment process. Initially, 198 patients were screened for eligibility during the study period. Out of this group, 130 patients (66%) fulfilled the inclusion criteria (eligible patients). Ultimately, 36 patients agreed to participate and successfully completed all study procedures (28% of eligible patients). Patients with a KPS of less than 70 were the primary reason for ineligibility. The by far most common reason for study refusal was distance to the training site being too far (47% of eligible patients)

A summary of the study flow is provided in Fig. 2. A total of 36 patients (58% male) with GBM were included in the analysis as of end of March 2023. Median age was 60 years (range 35–84; 95% CI 56.3–63.8), with 31% being 65 years or older.

For a comprehensive overview of baseline characteristics, current treatment and further relevant clinical information please refer to Table 1. We highlight that more than two-thirds of GBM patients experienced neurological impairments. Further, all patients completed combined chemoradiation prior to testing. Median time between completion and testing was 5 (IQR: 6.3) weeks. At testing, all participating patients were undergoing adjuvant chemotherapy, however testing was performed in treatment free intervals (except for one patient receiving temozolomide on a daily basis (100mg/day)).

**Table 1:** Patient characteristics prior to CPET

	Mean ± SD / n (%)
Patients	36 (100)
Socio-demographic	
Sex, male	21 (58)
Age, years	60.0 ± 11.1
≥ 65 yrs	11 (31)
Clinical information	
Karnofsky performance status (IQR)	90 (IQR: 20)
100	11 (31)
90	11 (31)
80	7 (19)
70	7 (19)
Body mass index (BMI)	25.0 ± 4.5
Smoking status	
Never	30 (83)
Quit	4 (11)
Currently	2 (6)
Tumor localization—laterality	
Right	15 (42)
Left	18 (50)
Both	3 (8)
Tumor localization—region	
Thalamus	2 (6)
Frontal	7 (19)
Temporal	4 (11)
Parietal	7 (19)
Occipital	1 (3)
Infratentorial	1 (3)
Multilobular	14 (39)
IDH-wildtype	36 (100)
MGMT promoter methylation (incl. weak methylation)	22 (61)
Neurological impairment (multiple answers possible)	25 (69)
Motor	8 (22)
Sensory	6 (17)
Visual	7 (19)
Speech	15 (42)
Epilepsy (tumor associated, taking anti-epileptic drug(s))	22 (61)
Treatment information	
Cranial surgery	36 (100)
Biopsy only	11 (31)
Partial resection (5–95% tumor resection)	12 (33)
Total resection (≥ 95% tumor resection)	13 (36)
Time post diagnosis, weeks (IQR)	14.5 (IQR: 6.9)
Time post radiotherapy, weeks (IQR)	5 (IQR: 6.3)
Current adjuvant treatment (after concurrent radiochemotherapy)	36 (100)
Temozolomide [18]	19 (52)
Lomustine-temozolomide combination [19]	15 (42)
Other (temozolomide and/or hydroxyurea)	2 (6)
Current dexamethasone treatment (administered ≤ 48h prior to testing)	16 (44)
Co-morbidities (permanent drug treatment; multiple answers possible)	20 (56)
Cardiovascular disease*	16 (44)
Diabetes	3 (8)
Others**	14 (39)
Relevant medical conditions (no permanent drug treatment; multiple answers possible)	
Allergies (incl. drug allergies and drug intolerances)	16 (44)
Orthopedic impairments	8 (22)
Thrombosis (tumor associated, treated & resolved) prior to testing	4 (11)
Others***	4 (11)

*Cardiovascular treatments include mono- and polytherapies (all cardiovascular drug classes, and combination therapies with diuretics, coagulation modulators, and lipid modulators)

** Others include hypo-/hyperthyreosis 6x, psychic disorders 4x, hormonal substitution (breast cancer in remission), prostate hyperplasia, restless legs syndrome, and hyperuricemia

*** Others include polyneuropathy 2x, psoriasis 1x, and chronic calcifying pancreatitis 1x

Of note: Five patients had malignant diseases prior to GBM; four of them were cured (kidney cancer (diagnosed 2005), prostate cancer 2x (diagnosed 2010 and 2019, resp.), colon cancer (diagnosed 2003)), and one patient was in remission (breast cancer (diagnosed 2020))

Regarding co-morbidities, the observed entities and numbers are not unusual (Table 1). Notably, we observed a relatively high number of thromboembolic events prior to testing. In one case, testing was postponed due to suspicion of thrombosis. The suspicion was later confirmed, and after treatment and clearance by the attending physician, the patient participated. Another patient was excluded from the study solely due to the presence of thrombosis discovered during initial examinations (not included in Table 1).

### Self-reported exercise

**Fig. 3:**
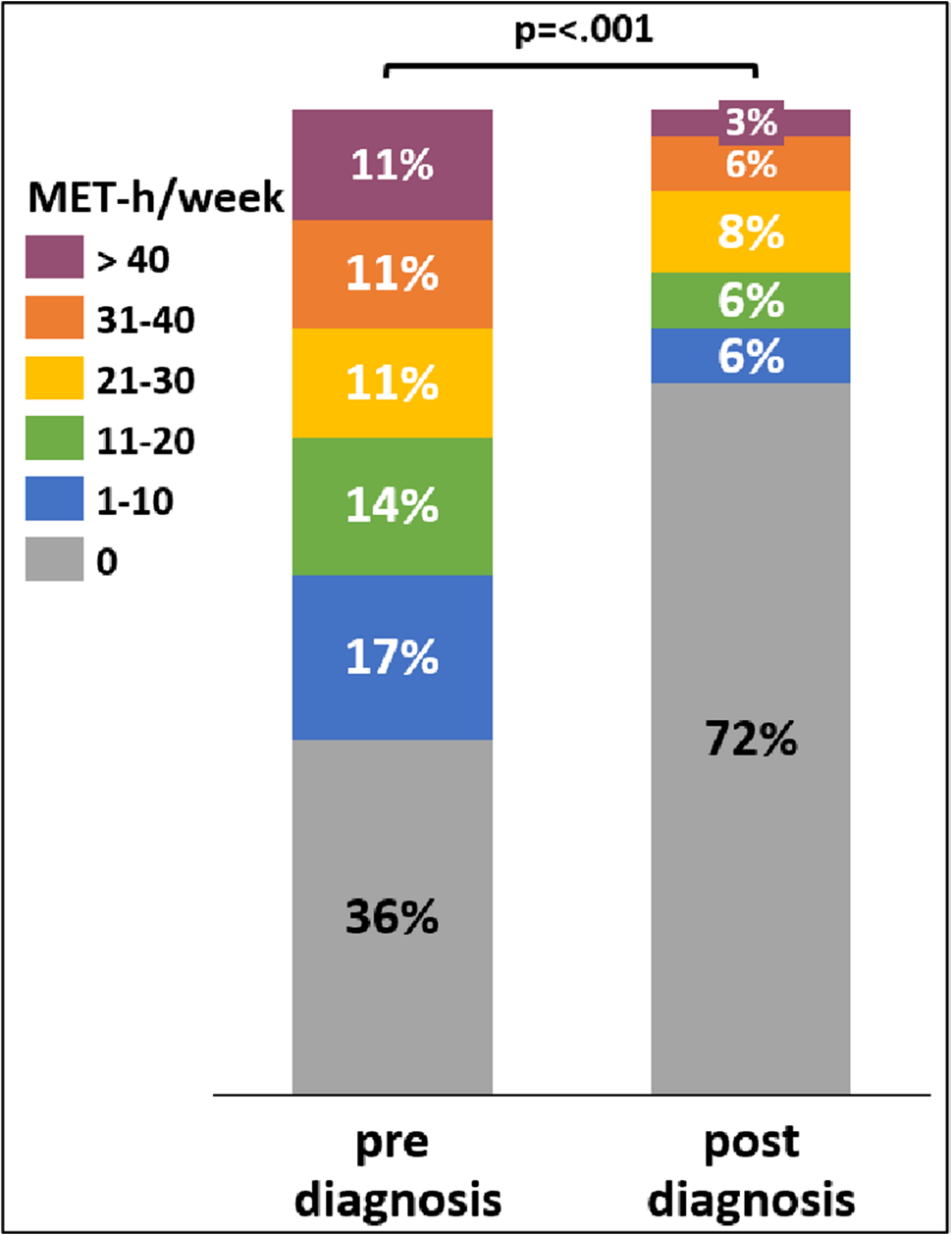
Self-reported exercise levels pre- and post-diagnosis. To determine the individual metabolic equivalents (MET) in hours per week, only systematic exercise was considered. The mean value pre-diagnosis was 15.8 MET-h/week and post-diagnosis decreased to 7.2 MET-h/week (p < 0.001; decrease of MET-hours per week: -54%). Suppl. Figure 2 provides a more detailed breakdown, allowing for a deeper understanding of changes in exercise patterns following diagnosis for both active and non-active patient groups

Before diagnosis, the median duration of systematic exercise (excl. walks) was 120 (IQR: 293) minutes per week (mean: 177 min/wk). After diagnosis, the duration of systematic exercise significantly decreased to a median of 0 (IQR: 83) minutes per week (p < 0.001) (mean: 83 min/wk). These data were converted into metabolic equivalents (METs) hours per week (see Fig. 3). When considering aerobic exercise and resistance exercise separately, it becomes apparent that a small proportion (19%) were found to meet national guidelines for aerobic exercise recommended for oncologic patients, i.e., either 150 min of moderate-intensity or 75 min of vigorous-intensity per week [20], as indicated by the activity data obtained from the questionnaires on physical activity behavior. Regarding national guidelines for resistance exercise (which recommend 2–3 sessions weekly targeting major muscle groups), only 6% of participants met the recommendation. Furthermore, the majority (44%) reported engaging in walks only, while 28% reported being inactive altogether.

### Feasibility

All patients achieved maximal exertion as shown in Table 2. Specifically, all patients reached over 80% of their age-predicted HRmax. Furthermore, RER exceeded 1.10 in 92% of patients (the minimum RER was 1.08, demonstrating consistent physiological response to the exercise stimulus). These values aligned with the subjective perceptions of the patients, reporting an average of 18.74 (range 16–20) on the Borg scale. 26 GBM patients (72%) fulfilled all five criteria for peak workload (Table 2). Notably, all patients reported to have neither (acute or chronic) respiratory symptoms nor respiratory disease. However, upon CPET, three participants (8%) had a (very) mild obstructive lung function, as indicated by a FEV1/FVC ratio below lower limit of normal (aligning with international guidelines), as assessed in the pre-testing spirometry and evaluated by the pulmonary physician. These participants did not self-describe specific respiratory symptoms at maximal workload. Regarding patient comfort, 4 patients (11%) found the mask used during the CPET procedure to be quite bothersome, however, not adversely affecting the overall execution.

**Table 2:** Criteria for reaching maximal exertion during CPET

Criteria for peak workload assessment [threshold criterion]	Values at maximum exertion (Mean ± SD/ Median (IQR))	Patients meeting threshold criterion at maximum exertion (%)
Heart rate, % of norm [21] [≥ 80% of norm]	96.46% ± 8.37%	100%
Respiratory exchange ratio [> 1.10]	1.22 ± 0.09	91.7%
Respiratory equivalent [> 35]	45.49 ± 6.98	94.3%
Lactate at test termination [> 5.0 mmol/l]	8.58 ± 2.32	93.9%
Perceived effort (Borg scale, 6–20) [≥ 17]	19 (2)	96.8%

Criteria were determined based on reference values provided by Kroidl et al. [16] and Löllgen & Leyk [15]. It was determined that peak performance is considered to be achieved only if at least one of these values was exceeded. Patients were encouraged to continue CPET until their individual point of exhaustion. In none of the patients, premature termination due to pathological changes or adverse events was necessary. The maximum predicted HR was calculated using the Tanaka’s Equation [21] (208—0.7 × age in years). While almost all participants reached peak lactate levels suggested for patients (> 5 mmol/l), two thirds even reached lactate levels > 8 mmol/l, which serves as a criterion for peak workload in healthy subjects [22]

### Safety

No participant except for one (3%) experienced any adverse events during testing at baseline. This patient experienced a minor adverse event (grade 1 according to CTCAE V5), characterized by a short post-test dizziness and temporary leg weakness causing a minor loss of balance (no fall). Within less than 3 min, the patient's condition fully normalized. No incidents of falls, epileptic seizures, or severe/moderate adverse events were reported.

### Cardiorespiratory fitness

On average, the participants' VO_2_peak (1750 ± 529 ml/min) was lower than the calculated normative values (p < 0.001) (Table 3). More precisely, 80% of patients were below the normative values for VO_2_peak. Similarly, the mean maximal workload achieved (130 ± 43 W) was also lower with 86% not meeting the expected normative values (p < 0.001). Likewise, the mean workload achieved at 75% of the HRmax (0.99 ± 0.38 W/kg BW) was found to be lower than the corresponding normative values (p = 0.027). The failure to reach the normative values applied equally to both sexes.

**Table 3:** Summary of cardiorespiratory fitness

Physical fitness parameter	Absolute values of GBM patients (Mean ± SD)	Percent achieved of individual predicted normative value (Mean ± SD)	P-value (GBM patients compared with normative value)
VO_2_peak [ml/min]	1750 ± 529	87% ± 16% (Gläser et al.) 86% ± 21% (Jones et al.)	p < 0.001 p < 0.001
VO_2_peak [ml/kg BW/ min]	22.9 ± 5.4	90% ± 27% (Jones et al.)	p = 0.005
Maximal workload [W]	130 ± 43	79% ± 18% (Gläser et al.) 82% ± 22% (Jones et al.)	p < 0.001 p < 0.001
PWC 75% [W/kg BW]	0.99 ± 0.38	90% ± 29% (Finger et al.)	p = 0.027

To determine normative values, established equations proposed by Gläser et al. [17] and Jones et al. (referenced in [15]) for calculating VO2peak and maximal workload were utilized. The PWC values achieved at 75% of HRmax were compared to normative data derived from the study by Finger et al. [23] as none of the other cited authors provided normative data for PWC. Further comparisons with normative data (Wasserman, Haber, Hansen, Jones, and Cooper), including VO2peak per kilogram of bodyweight, can be found in the Supplement (see Suppl. Table 3)

As expected, we observed a clinically meaningful association between KPS and CPET performance, evident in both maximal workload and VO_2_peak (refer to Suppl. Figure 3A and B). Likewise, albeit to a lesser extent, a meaningful association was found between different activity level groups and CPET performance, as shown in Suppl. Figure 3C and D.

## Discussion

We report on max. CPETs conducted in 36 consecutive GBM patients undergoing chemotherapy. Three main findings can be inferred:

Firstly, max. CPET in GBM patients was feasible and safe, despite numerous challenges, i.e., history of cranial surgery, completion of radiochemotherapy, ongoing chemotherapy, and various impairments among GBM patients. Additionally, some patients had a medical history of tumor-associated epilepsy and various other comorbidities. Further, most patients did not exercise in the period leading up to testing and were unaccustomed to physical exertion (compare Fig. 3). Despite these challenges, patients were able to reach peak workload. No relevant side effects occurred during CPET, including no falls or epileptic seizures. Just in one case, a short, transient, minor post-test dizziness occurred, with no intervention required. These results align with our previous work, using less sophisticated assessment in a retrospective design [7]. As of now, apart from one study [24], there have been no known investigations in which GBM patients were subjected to maximal stress during CPET. Nevertheless, in this particular study, some patients were unable to reach their maximal cardiovascular function due to fatigue or leg weakness. On a related note, Culos- Reed et al. concluded that the assessment of aerobic capacity by means of VO_2_peak was impractical [25]. Gehring et al., by contrast, conducted a max. CPET with clinically stable grade 2 and 3 glioma patients only (stable for a minimum of six months prior study entry) and did not report any adverse events [26]. Submaximal testing with brain tumor patients using a cycle ergometer was also performed in certain studies, where exercise was limited up to 80% HRmax [27] or terminated upon reaching the second ventilator threshold [25, 28]. These approaches were chosen to address concerns about the vulnerability of the patient population. Nevertheless, based on our results, we find it reasonable to assume that Watt-max testing can be safely conducted even with GBM patients undergoing chemotherapy. Our findings are consistent with safely performed max. CPET studies in other malignancies, including breast cancer, prostate cancer, non-small cell lung cancer, small cell lung cancer, Hodgkin's lymphoma, testicular cancer, head and neck cancer, and adult survivors of childhood cancer [10–13, 22,  29, 30].

Nevertheless, we would like to highlight some safety measures and practical advice derived from our testing experience. Since half of GBM patients are 60 years or older, they often present with major comorbidities, such as cardiovascular diseases (here: 44%, compare Table 1), which are more prevalent in older age groups [9]. Therefore, special attention should be given during testing to ensure safety of these individuals. Additionally, it is crucial to consider that GBM patients are at a higher susceptibility to prothrombotic events [31–33]. In our patient cohort, 11% of individuals experienced thrombosis since their initial diagnosis. As thrombosis poses a serious risk for sudden embolic, potentially life-threatening events during intensive exercise, it is advisable to thoroughly preclude thrombosis before exercise testing and conducting training programs.

Secondly, the analysis of cardiorespiratory fitness in comparison to established normative data revealed reduced fitness levels in GBM patients post combined radiochemotherapy. The average lower levels of cardiorespiratory fitness observed in our participants align with findings from other studies in glioma patients [34–37]. These studies reported patients achieving 56–79% of predicted norm, partly attributed to the testing protocols with submaximal termination thresholds as indicated by lower RER values (RER ≤ 1.0) compared to our study. The reduced fitness levels after intensive treatment, including neurosurgery and radiation, highlight the need for exercise programs specifically designed to improve cardiorespiratory fitness in this patient population.

Higher physical fitness is associated with reduced physical fatigue [34], less severe symptom burden, and improved QoL [5] in brain tumor patients. Interestingly, despite undergoing surgical treatment, cerebral radiation, and chemotherapy, participants of our study exhibited physical conditions that were less compromised than expected. This could be due to a subset of patients who continued regular PA even post-diagnosis (compare Suppl. Figure 2C). Those maintaining some level of systematic exercise, even at a lower intensity, demonstrated clinically meaningful better fitness outcomes (compare Suppl. Figure 3C and D).

This leads to our third major observation, focusing on the development of the patients’ exercise behavior throughout tumor trajectory. Self-reported exercise patterns before and after diagnosis indicated a significant reduction, ranging from decrease to complete cessation of PA. This is noteworthy, considering previous research consistently demonstrating numerous positive effects of exercise for cancer patients, regardless of disease phase or type of PA employed [4]. While some patients continue endurance exercises, there is an almost complete lack of resistance training among them, a behavior also observed in other exercise studies involving brain tumor patients [5, 25, 35]. In contrast, Jones et al. found that the majority of exercise behavior outcomes remained stable in glioma patients (all grades), suggesting their ability to engage in PA during adjuvant treatment independently [38]. However, as reported here, this is not the case in a pure GBM population. Despite apparent interest in participating in a sports program — as evidenced by their enrollment in our sports study – most patients in this study encountered challenges in independently engaging in systematic exercise (compare Fig. 3). This decline in exercise participation might be attributed to feelings of fear, uncertainty, and possible harm given their medical condition. On a related note, Halkett et al. emphasized managing symptoms, organizational issues, and difficulties with engaging in their exercise intervention as barriers to exercise, while also concluding that patients' and carers' perceptions of exercising with brain tumor patients under therapy were generally very positive [39]. Addressing these fears and barriers to exercise at large could help patients maintain an active lifestyle, thereby contributing to enhancing their overall QoL.

In addition to the three main results, we would like to discuss practical testing recommendations. These are offering insights for test conduct derived from our gathered experiences and are included in the Supplement, as they are not directly related to the research questions.

The main limitation of our study relates to potential selection bias due to inclusion and exclusion criteria. Participants included were all interested in participating in a sports program and had a KPS of at least 70, possibly indicating better physical fitness outcomes even in cases of severe disease compared to those not genuinely interested in exercise. Further, although we carefully collected exercise behavior prior and post diagnosis, we did not systematically assess patients’ longitudinal exercise history throughout their lives.

## Conclusion

In conclusion, the findings of this study suggest that max. CPET is a safe and viable method for reliably assessing cardiorespiratory fitness and exercise capacity in certain GBM patients. The objective data gathered through the procedure employed can contribute to the understanding of the fitness status of this patient population and assist in developing tailored exercise programs and monitoring progress. Additionally, our results indicate that GBM patients undergoing adjuvant chemotherapy exhibit reduced levels of cardiorespiratory fitness, highlighting the need for individualized exercise programs to enhance overall physical fitness and QoL.

### Supplementary Information

Below is the link to the electronic supplementary material.

Supplementary file1 (DOCX 248 KB)

## Data Availability

We will make our research data available upon reasonable request. Researchers interested in accessing the data may contact the corresponding author. Access will be provided in accordance with ethical and legal guidelines, subject to necessary approvals.

## Abbreviations

CPET: Cardiopulmonary exercise testing
CTCAE: Common terminology criteria for adverse events
GBM: Glioblastoma multiforme
HR: Heart rate
IQRs: Interquartile ranges
KPS: Karnofsky performance status
MET: Metabolic equivalents
PA: Physical activity
PWC: Physical work capacity
QoL: Quality of life
RER: Respiratory exchange ratio
SD: Standard deviations
VO_2_peak: Peak oxygen consumption
WHO: World Health Organization
