# Correction to: Maximal cardiopulmonary exercise testing in glioblastoma patients undergoing chemotherapy: assessment of feasibility, safety, and physical fitness status

**DOI:** 10.1007/s11060-024-04686-3

**Published:** 2024-04-24

**Authors:** Johanna Jost, Klaus Völker, Ralf Brandt, Walter Stummer, Steffi Urbschat, Ralf Ketter, Dorothee Wiewrodt, Rainer Wiewrodt, for the MMH Trial Investigators, Maren Kloss, Nora Hansel, Irmtraud Früchte, Ross Julian, Lothar Thorwesten, Joachim Gerß, Andreas Faldum, Joachim Oertel, Philipp Lepper, Kathleen Jetschke, Sylvia Rekowski, Carolin Weiss Lucas, Sophia Kochs, Freerk Baumann

**Affiliations:** 1 Department of Neurosurgery, University Hospital, University Münster, Münster, Germany; 2 Institute of Sports Science, University Hospital, University Münster, Münster, Germany; 3 Department of Neurosurgery, University Hospital, Saarland University, Saarbrücken, Germany; 4 Pulmonary Research Division, Department of Medicine A, University Hospital, University Münster, Münster, Germany

**Correction to: Journal of Neuro-Oncology**

10.1007/s11060-024-04629-y

In this article, the names of investigators Ross Julian, Lothar Thorwesten and Joachim Oertel were omitted in the data captured for the MMH Trial Investigators.

The original article has been corrected.

