# Supplementary material for: Maximal cardiopulmonary exercise testing in glioblastoma patients undergoing chemotherapy: assessment of feasibility, safety, and physical fitness status
Source: J Neurooncol. 2024 Apr 1;168(1):35–45. doi: 10.1007/s11060-024-04629-y (PMC11093868; doi:10.1007/s11060-024-04629-y)
Supplement: Supplementary file 1 — Supplementary file1 (DOCX 248 KB) [file 11060_2024_4629_MOESM1_ESM.docx]

**SUPPLEMENT**

**Supplementary Content 1: Additional information on the SHIP-study (Original Abstract of Gläser et al. [1])**

“The interpretation of gas exchange measured by cardiopulmonary exercise testing (CPET) depends on reliable reference values. Within the population based Study of Health in Pomerania (SHIP) CPET was assessed in 1706 volunteers. The assessment based on symptom limited exercise tests on a bicycle in a sitting position according to a modified Jones protocol. CPET was embedded in an extensive examination program. After the exclusion of active smokers and volunteers with evidence of cardiopulmonary and musculoskeletal disorders the reference population comprised 616 healthy subjects (333 women) aged 25 to 85 years. Reference equations including upper and/or lower limits based on quantile regression were assessed. All values were corrected for the most important influencing factors. This study provides reference equations for gas exchange and exercise capacity assessed within a population in Germany.”

|  | Men | | | | Women | | | |
| --- | --- | --- | --- | --- | --- | --- | --- | --- |
| Age group | 25-44 years | 45-64 years | 65-80 years | Total | 25-44 years | 45-64 years | 65-80 years | Total |
| N | 120 | 124 | 39 | 283 | 151 | 156 | 26 | 333 |
| Height [cm] | 180  (177; 184) | 176  (171; 180) | 170  (167; 177) | 177  (172; 181) | 166  (163; 170) | 164  (160; 167) | 161  (157; 165) | 165  (161; 169) |
| Weight [kg] | 83  (76; 93) | 84  (76; 94) | 79  (74; 89) | 83  (76; 93) | 65  (58; 75) | 69  (62; 78) | 69  (58; 78) | 67  (60; 77) |
| BMI [kg/m²] | 26  (24; 28) | 28  (25; 30) | 27 (25; 30) | 27  (25; 29) | 23  (21; 27) | 25  (23; 28) | 27  (23; 29) | 25  (22; 28) |
| VO_2_peak [ml/min] | 2900  (2515; 3273) | 2389  (2129; 2650) | 1885  (1660; 2300) | 2500  (2154; 2955) | 1750  (1550; 2043) | 1600  (1429; 1803) | 1435  (1240; 1544) | 1650  (1473; 1900) |

**Supplementary Table 1. SHIP-study population characteristics.** Participant characteristics of the SHIP-study by age groups and separated by gender. Values as 50th (25th; 75th) percentiles (taken from Gläser et al., 2013 [1])

| Parameter | Quantiles | Men | Women |
| --- | --- | --- | --- |
| Maximal workload [W] | 0.05  0.10  0.50 | –202,126 – 0,8136 * A + 2,2017 * H–0,0236 * W  –50,668 – 1,0416 * A + 1,3858 * H + 0,2037 * W  –103,512 – 1,5576 * A + 2,2114 * H–0,1198 * W | 24,479 – 0,7804 * A + 0,5196 * H + 0,3733 * W  10,425 – 0,6341 * A + 0,6871 * H + 0,1785 * W  –80,628 – 0,7698 * A + 1,4038 * H + 0,2873 * W |
| VO2peak [ml / min] | 0.05  0.10  0.50 | 533,071 – 15,0769 * A + 7,1869 * H + 9,4892 * W  1761,773 – 17,1974 * A + 1,2277 * H + 10,1231 * W  254,761 – 22,6925 * A + 17,2463 * H + 4,4114 * W | 531,387 – 7,7033 * A + 2,9842 * H + 9,1722 * W  389,056 – 7,2668 * A + 4,6393 * H + 7,9446 * W  –54,739 – 9,8085 * A + 9,9172 * H + 8,0557 * W |

**Supplementary Table 2. SHIP-study reference value formulas.** This table contains reference value formulas for maximal workload and VO_2_peak obtained during SHIP-1, based on non-smoking participants without detectable cardiopulmonary morbidity within the framework of the study program. The formulas each include the 50th percentile as well as upper and lower reference value limits (5th/10th and 90th/95th percentiles). Influencing factors such as age, height, and weight are included as continuous variables in the reference value formulas (taken from Gläser et al., 2013 [1]).

| **Investigators** | **Percent predicted [%]**  **Mean ± SD** | | |
| --- | --- | --- | --- |
|  | **VO_2_peak**  **[ml / min]** | **VO_2_peak**  **[ml / kg BW / min]** | **Maximal workload [W]** |
| **Cooper et al. [2]**  (1978, USA) | - | 99% ± 25% | - |
| **Hansen et al. [3]**  (1984, USA) | 79% ± 17% | - | - |
| **Jones et al. [4]**  (1985, Canada) | 86% ± 21% | 90% ± 27% | 82% ± 22% |
| **Wasserman et al. [5]**  (1999, USA) | 94% ± 19% | - | 105% ± 35% |
| **Haber et al. [6]**  (2013, Austria) | - | - | 87% ± 21% |
| **Gläser et al. [1]**  (2013, Germany) | 87% ± 16% | - | 79% ± 18% |

**Supplementary Table 3. Comparison of achieved physical fitness values of GBM patients with normative values of healthy subjects.** Various cohorts were applied. All normative equations used are adjusted for sex, age, height, and/or weight and were derived from Kroidl et al. 2015 [7].

There are no standardized and binding reference values for CPET parameters. However, in this study, we chose to utilize established equations proposed by Gläser et al. [1] for further comparisons and analyses of VO2peak and maximal workload. This decision was based on the fact that our testing procedures closely resembled the protocols used in the SHIP study [1], including seated ergometer tests with an incremental CPET protocol, applied to patients up to the age of 84. Furthermore, 616 German healthy subjects were included in the SHIP study making it a representative population, and the generated normative values closely align with those of other authors, such as Wasserman et al. [5]. For these reasons, we considered the SHIP normative values [1] to be the best reference for this study.

| **Type of Physical Activity** | **MET** | **Intensity** |
| --- | --- | --- |
| Yoga | 3 | mild |
| Pilates | 3 |  |
| Aquarobic | 5 | moderate |
| Nordic Walking | 5 |  |
| Resistance Training (moderate) | 5 |  |
| Soccer (leisure sports) | 7 | vigorous |
| Running (jogging, general) | 7 |  |
| Cycling stationary (150 W) | 7 |  |
| Swimming (light effort) | 7 |  |

**Supplementary Table 4. MET assignment to types of physical activities.** These MET values were determined based on the guidelines provided by Ainsworth et al., 1993 [8]. All types of activities reported by the participating patients are listed in the table.


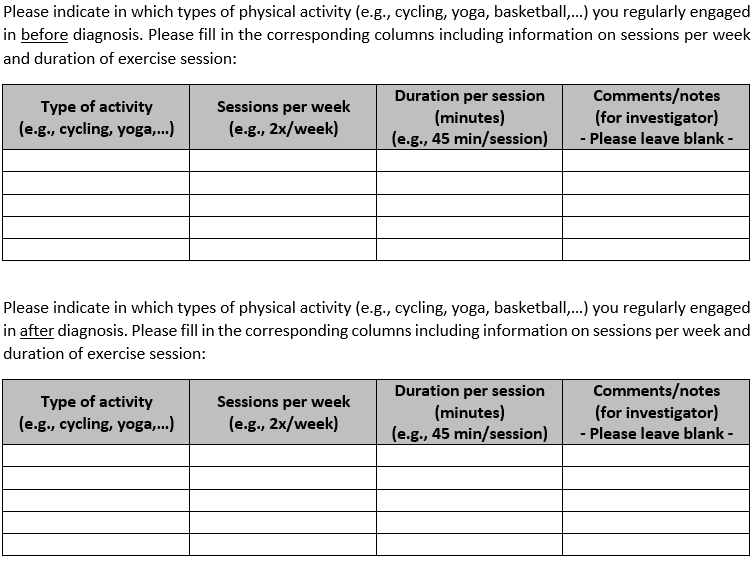


**Supplementary Figure 1: Patient questionnaire on physical activity behavior.** This figure presents a modified, translated version of the applied patient questionnaire distributed to patients as part of the screening procedure and filled out jointly with the examiner. It includes the type, frequency, and duration of exercises performed. In cases of uncertainty regarding the intensity, the examiner requested further information (e.g., 'Did you engage in leisure or competitive soccer?'). Subsequently, the responses were converted to MET-hours per week (as described in Supplementary Table 4)."


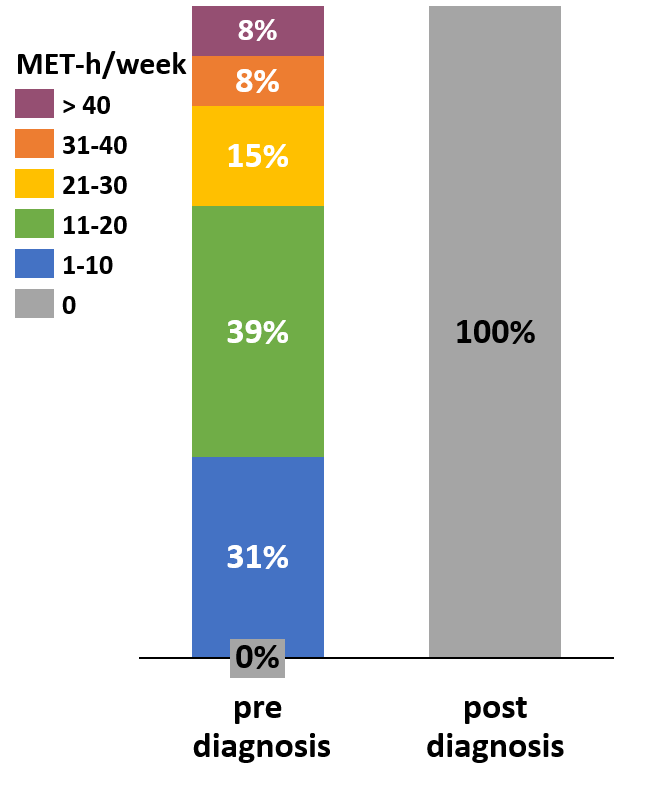

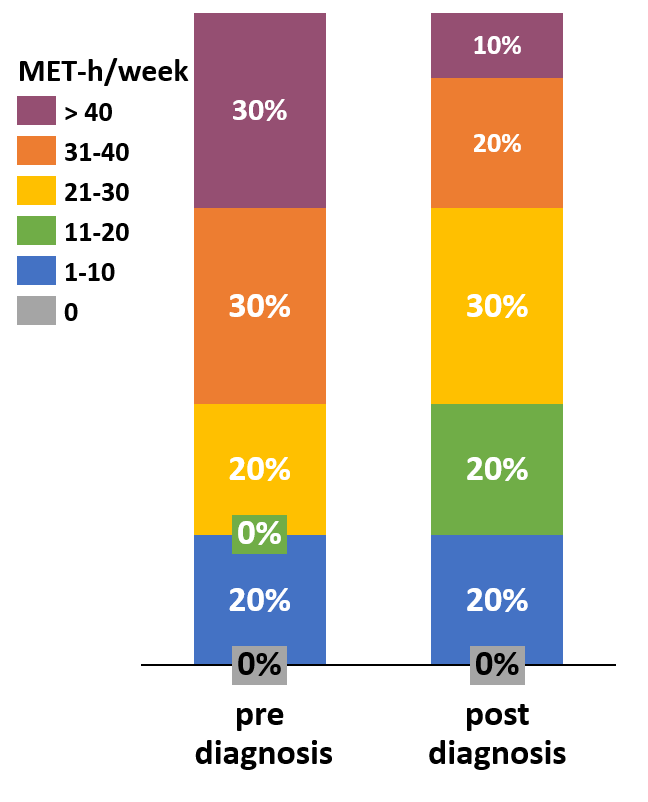

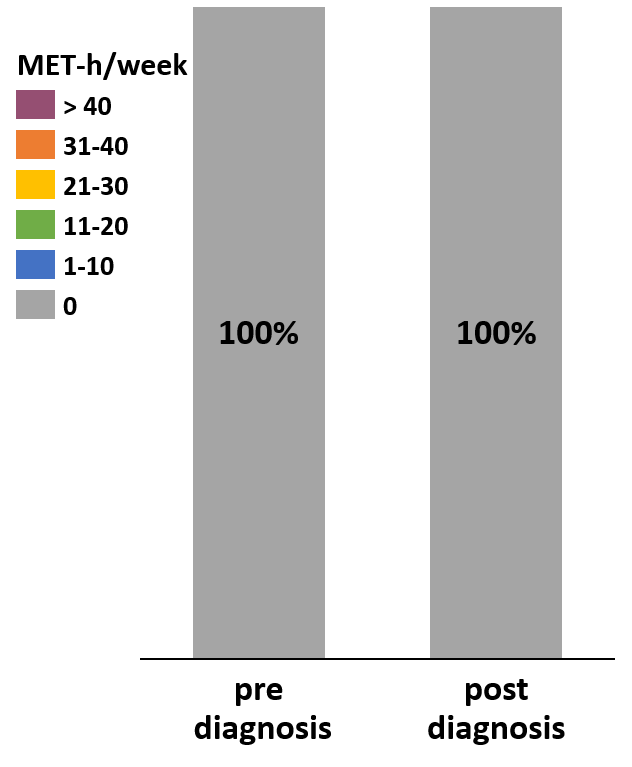


**Preoperative active patients with ongoing activities after surgery (n=10)**

**“Stay-active patients”**

**Preoperative inactive patients**

**(n=13)**

**“All-inactive patients”**

**Preoperative active patients, but inactive after surgery (n=13)**

**“Quitters”**

**A**

**B**

**C**

**Supplementary Figure 2: Distribution of self-reported exercise by activity levels pre- and post-diagnosis.** Shown are three activity groups of GBM patients (n=36): (A) All-inactive patients prior to diagnosis remained inactive after diagnosis, without initiating any exercise on their own (walks are excluded). (B) Among those who stopped exercising after diagnosis (“quitters”), the average pre-diagnosis exercise level was 16.7 MET-hours per week (decrease of MET-hours per week: -100%). (C) “Stay-active patients” decreased from an average pre-diagnosis exercise level of 35.2 MET-hours per week to 26.1 MET-hours per week post-diagnosis (decrease of MET-hours per week: -26%).


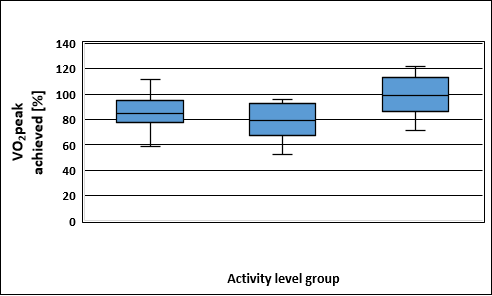

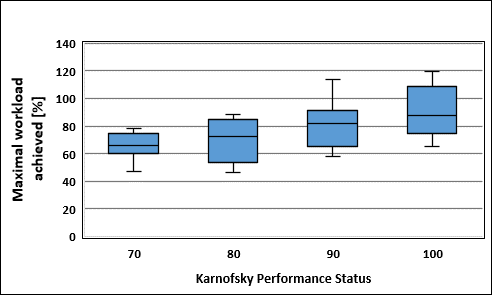

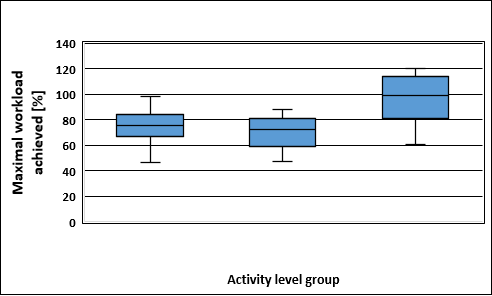

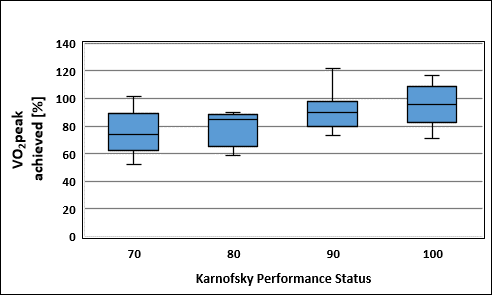


**Stay-active patients**

**Quitters**

**All-inactive patients**

**All-inactive patients**

**Stay-active patients**

**Quitters**

**p=.035** (all comparisons)

**p<.01** (all comparisons)

**p<.02** (all comparisons)

**p<.01** (all comparisons)

**D**

**C**

**B**

**A**

**Supplementary Figure 3: Influence of Karnofsky Performance Status (A, B) and exercise behavior (C, D) on CPET outcomes.** All comparisons revealed a clinically meaningful impact of both parameters on CPET outcomes. To further elucidate this finding, we conducted a logistic regression analysis, incorporating age, sex, dexamethasone intake, BMI, cardiovascular medications, KPS, and exercise behavior for both variables (maximal workload (A, C) and oxygen uptake, i.e., VO_2_peak (B, D). Both regression models confirmed that KPS and exercise behavior are independent variables for CPET performance (p<.001 for both parameters across the two models).

**Supplementary content 2: Practical recommendation on CPET testing**

In addition to the three main results of maximal CPET-testing, we would like to discuss practical testing recommendations derived from our gathered experiences.

1. **Lactate levels during peak exertion.** It is essential to note that in patients with multiple treatments, including current chemotherapy, may have lower peak lactate levels (mean here: 8.58mmol/l) compared to healthy individuals. A possible reason for lower peak lactate levels in GBM patients could be a reduced muscle mass [9, 10] or myopathy due to extended periods of immobility and hospitalization. This is in line with the recommendations of Löllgen and Leyk, who also suggest lower peak lactate values for patients (> 5mmol/l) in comparison to healthy subjects (> 9mmol/l) [11].
2. **CPET protocol.** In this study, we chose 25-Watt-increments to maintain comparability with data collected in our previous work [12]. During the presented study, it became evident that 25-Watt increments used in the exercise testing proved too high for some GBM patients, particularly for those who did not reach 100 Watts at their peak performance. Therefore, we would recommend opting for a ramp or “quasi-ramp” protocol with small increments (e.g., 10 Watts/min) as has been suggested for testing endurance performance in (oncologic) patients [13] for patients with lower fitness levels. Alternatively, one could follow a similar approach to Jones et al., in which the initial workload and subsequent increments were customized based on the glioma patients' medical history and metabolic responses to exercise within the first minute [10].
3. **Individual determination of training intensity**. CPET results upon study entrance were utilized for the development of the patients’ training plan. The training modulation was primarily based on HR parameters [12]. Thus, CPET was essential in capturing the patients’ authentic HRmax values. In addition, the maximal workload achieved can serve as an orientation for individual determination of training intervals, too. **At training commencement**, based on our experience, we recommend to limit the intensity for the most demanding training interval to 80-90% of CPET peak performance (which normally accounts for 80-90% of HRmax) achieved during testing.

Further detailed insights and results following training intervention will be presented in the main paper of the MMH study upon its completion.

**REFERENCE LIST FOR SUPPLEMENT**

[1] Gläser S, Ittermann T, Schäper C et al. Referenzwerte für die Spiroergometrie – Ergebnisse der Study of Health in Pomerania (SHIP*) [Reference Values for Spiroergometry - Results from the Study of Health in Pomerania (SHIP)]*. Pneumologie 2013; 67: 58-63.

[2] Cooper KH, Pollock ML, Wilmore JH et al. Health and fitness through physical activity. New York: John Wiley; 1978

[3] Hansen JE, Sue DY, Wasserman K. Predicted values for clinical exercise testing. Am Rev Respir Dis. 1984;129(pt 2): S49–S55.

[4] Jones NL, Makrides L, Hitchcock C, Chypchar T, McCartney N. Normal standards for an incremental progressive cycle ergometer test. Am Rev Respir Dis. 1985 May;131(5):700-8.

[5] Wasserman K, Hansen JE, Sue DY, Casaburi R, Whipp BJ. Principles of Exercise Testing and Interpretation: Including Pathophysiology and Clinical Applications. 3rd ed. Philadelphia (PA): Lippincott Williams & Wilkins; 1999.

[6] Haber P. Lungenfunktion und Spiroergometrie. 3. Aufl. Wien: Springer; 2013. Auf der Grundlage von Niederberger M et al. Acta Medica Austriaca 1975; 2: 33-48, und neuerlich 2008 in Wonisch M et al. Praxisleitlinien Ergometrie. J f Kardiologie 2008; 15 (Suppl. A) *[Pulmonary Function and Spiroergometry. 3rd Edition. Vienna: Springer; 2013. Based on Niederberger M et al. Acta Medica Austriaca 1975; 2: 33-48, and more recently in Wonisch M et al. Guidelines for Exercise Testing. J Cardiology 2008; 15 (Suppl. A).]*

[7] Kroidl RF, editor. Kursbuch Spiroergometrie: Technik und Befundung verständlich gemacht [Coursebook Spiroergometry: Technology and Interpretation Made Understandable]. 3rd ed. Stuttgart, New York, NY: Thieme; 2015. 526 p. German.

[8] Ainsworth BE, Haskell WL, Leon AS, et al. Compendium of physical activities: classification of energy costs of human physical activities. Med Sci Sports Exerc. 1993;25(1):71-80.

[9] Steindl A, Leitner J, Schwarz M, et al. Sarcopenia in Neurological Patients: Standard Values for Temporal Muscle Thickness and Muscle Strength Evaluation. J Clin Med. 2020;9(5). doi:10.3390/jcm9051272 Cited in: PubMed; PMID 32354003.

[10] Jones LW, Friedman AH, West MJ, et al. Quantitative assessment of cardiorespiratory fitness, skeletal muscle function, and body composition in adults with primary malignant glioma. Cancer. 2010;116(3):695–704. doi:10.1002/cncr.24808 Cited in: PubMed; PMID 20029975.

[11] Löllgen H, Leyk D. Exercise Testing in Sports Medicine. Dtsch Arztebl Int. 2018;115(24):409–16. doi:10.3238/arztebl.2018.0409 Cited in: PubMed; PMID 29968559.

[12] Jost J, Müther M, Brandt R, et al. Conceptual development of an intensive exercise program for glioma patients (ActiNO): summary of clinical experience. J Neurooncol. 2023;163(2):367–76. doi:10.1007/s11060-023-04354-y Cited in: PubMed; PMID 37306887.

[13] Scharhag-Rosenberger F, Becker T, Streckmann F, Schmidt K, al e. Studien zu körperlichem Training bei onkologischen Patienten: Empfehlungen zu den Erhebungsmethoden [Studies on Physical Training in Oncological Patients: Recommendations for Assessment Methods]. Dtsch Z Sportmed. 2014;2014(11):304–13. doi:10.5960/dzsm.2014.148. German.
